# Mapping the current trends and hotspots of vascular cognitive impairment from 2000–2021: A bibliometric analysis

**DOI:** 10.1111/cns.14026

**Published:** 2022-11-22

**Authors:** Xu Han, Jian Zhang, Sifan Chen, Weifeng Yu, Yan Zhou, Xiyao Gu

**Affiliations:** ^1^ Department of Radiology, Department of Anesthesiology, Renji Hospital Shanghai Jiao Tong University School of Medicine Shanghai China

**Keywords:** bibliometric analysis, Citespace, keywords co‐occurrence and burst keywords, small vessel disease, vascular cognitive impairment

## Abstract

**Aims:**

To visualize the trends and hotspots in the scientific research related to vascular cognitive impairment (VCI) quantitatively and qualitatively.

**Methods:**

Cross‐sectional bibliometric analysis of publications that related to VCI was conducted. Publications were found by searching in the Web of Science Core Collection database (WoSCC) – Edition: Science Citation Index Expanded (SCI‐Expanded) from January 2000 to December 2021. Publication type was restricted to article and review in the English language. The downloaded data were screened and analyzed in January 2022.

**Results:**

In total, 16,264 publications were identified, with a steady increase in annual publications. The United States was the leading country in VCI research regarding publication numbers and national influence. National Institute of Aging had the highest influence among all the institutes in the field of VCI. Philip Scheltens was the most active author. The top five active authors' publications focused on pathobiology, neuroimaging standards, risk factors, prevention, and the standard diagnosis of vascular dementia (VaD). A co‐cited publication clustering resulted in 19 main clusters, and the prevention, blood–brain barrier, cholesterol, cerebral amyloid angiopathy, and VaD were the top 5 clusters. Moreover, burst keywords detection revealed that the “small vessel disease” is the current hotspot in the field of VCI.

**Conclusions:**

This bibliometric analysis mapped the overall research structure of VCI and analyzed the current research trends and hotspots for future studies orientation. Neuroimaging, risk factors detection, and pathobiology are the current trends in VCI research. Small vessel disease and its mechanisms are the current hotspots of VCI research.

## Graphical abstract

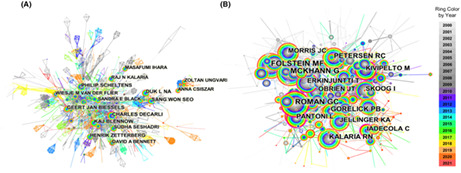

## 1 INTRODUCTION

The term “vascular cognitive impairment (VCI)” was introduced at the beginning of the third millennium for the first time. VCI comprises a broad spectrum of cognitive disorders, from mild cognitive impairment (MCI) to vascular dementia (VaD) caused by hemorrhage, ischemia, vascular etiologies alone or in combination with neurodegeneration and Alzheimer's disease (AD).^1^, ^2^ Formerly, AD was the most studied topic in the field of cognitive impairment or dementia; however, recent studies robustly revealed that vascular diseases are the main driver of the global burden of dementia.^3^ After including mixed pathology dementia and white matter hyperintensities (WMHs), VCI accounts for 50% to 70% of dementias.^1^ It is increasingly supported by recent findings that cerebrovascular pathology is the most important contributor to dementia, which enormously affects patients' quality of life.^4^, ^5^

The diagnosis of VCI needs a comprehensive clinical judgment corroborated by neuropsychological tests, and evidence of vascular pathology in neuroimaging, such as lacunar infracts, white matter hyperintensities, or microbleeds.^1^ The heterogeneity of the cerebrovascular pathologies and their clinical manifestations made it difficult to achieve broadly applicable diagnostic criteria. The International Vascular Impairment of Cognition Classification Consensus Study (VICCS‐1 and 2) has recently classified the diagnostic criteria for VCI and VaD into mild or major. Accordingly, four VCI subtypes were defined, including post‐stroke dementia, subcortical ischemic vascular dementia, multi‐infract (cortical) dementia, and mixed dementia.^6^ VICCCS‐2 recommended neuroimaging with magnetic resonance imaging (MRI) as a gold standard for diagnosing VCI.^7^, ^8^ Neuroimaging is used to assess the extent, location, and type of vascular lesions. MRI protocol should include T1‐weighted imaging to detect lacunar infracts and fluid‐attenuated inversion recovery (FLAIR) sequences to detect WMHs. In addition, susceptibility‐weighted imaging (SWI) detects microbleeds and superficial siderosis. Diffusion tensor imaging (DTI) can detect microinfarcts and abnormal changes in the white matter tracts. Assessment of β‐amyloid deposition by positron emission tomography (PET) scan, and non‐invasive assessment of the cerebral vasculature by carotid ultrasound or MR angiography are also suggested.^9^ There is no standardized neuropsychological test, and different research groups and clinical centers use various protocols. The cognitive evaluation should include an extensive examination of executive functions, memory, language, visuospatial functioning, attention, and mental speed. Montreal Cognitive Assessment (MoCA) is increasingly used to assess cognitive domains frequently impaired in patients with VCI. It can detect a more‐subtle decline in cognitive function than the Mini‐Mental State Examination (MMSE).^10^, ^11^, ^12^ The definite diagnosis of VCI is based on neuropathology. However, neuropathology cannot connect the symptoms to the disease and is performed after death or years after the onset of symptoms. Therefore, an accurate diagnosis of VCI needs further investigation.^3^

Several cerebrovascular pathologies are linked to VCI, including cerebral small vessel disease (SVD), large artery atherosclerosis, brain hemorrhages, cardioembolism, and other less common etiologies of stroke.^13^, ^14^ SVD is characterized by arteriolosclerosis and lacunar infarcts. SVD causes cortical and subcortical microinfarcts, and is the most frequent etiology of cognitive impairment.^15^, ^16^ A prospective screening study showed that seven pathologies, including large infracts, lacunar infracts, microinfarcts, myelin loss, arteriolosclerosis, leptomeningeal cerebral amyloid angiopathy (CAA), and perivascular space dilation, which were mostly associated with cognitive impairment.^17^ Vascular pathologies, such as arteriolosclerosis, atherosclerosis, and CAA, are common in older age and crucial for VCI. Therefore, incorporating of routine screening tests for these vascular pathologies into the neuropathological assessment of dementia may be rational.^18^, ^19^ Despite considerable progress in deciphering the link between cerebrovascular disease and cognitive impairment, still, there is more to be known.^9^

The bibliometric analysis applies statistical methods to analyze quantitative data about scientific activities. It generates an organizational knowledge structure by processing details of references such as journals, authors, institutions, etc.^20^ However, the quantitative methodology has been rarely used in the field of VCI. In this study, we analyzed VCI studies to evaluate research trends and potential hot spots between 2000 and 2021 worldwide. We also predict research trends in this field over the next few years.

## 2 METHODS

### 2.1 Search strategy and data collection

The publications related to vascular cognitive impairment were searched in the Web of Science Core Collection database (WoSCC) ‐ Science Citation Index Expanded (SCI‐Expanded) Editions. Search query was applied as ((TS = vascular cognitive impairment) OR (TS = vascular dementia)) from January 1, 2000 to December 31, 2021. Publication type was confined to article and review articles published in English. Full records, including titles, authors, keywords, country, institution, and references of each publication were collected on January 7, 2022, to prevent redundancy from frequent updates to the database. The data downloaded was screened by Xu Han and Jian Zhang to confirm the relationship on the topic.

All data were converted to plain text format by the WoS tool, then imported to CiteSpace V5.8.R3, 64‐bit (Drexel University, Philadelphia, PA, USA) for further bibliometric analysis.

### 2.2 Bibliometric analysis

**FIGURE 1:**
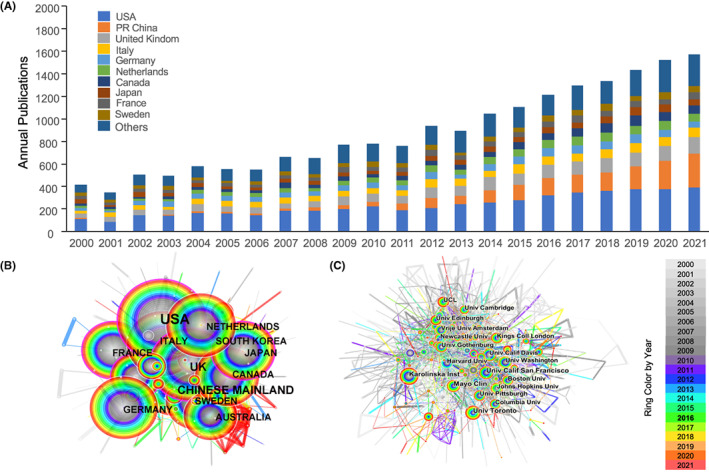
Annual publications and growth trend of top 10 and the rest countries/regions on vascular related cognitive impairment research from 2000–2021 (A). The network map of collaborating countries/regions (B) and institutions (C) in vascular cognitive impairment research.

The following characteristics were described: annual publications and growth trends, countries/regions, institutions, authors, co‐cited authors, clustered networks of co‐cited references and keywords, and keywords with the highest citation bursts.^21^ Data were mostly visualized by the clustering of CiteSpace, and the annual publications and growth trend (Figure 1) were generated by Microsoft Excel 2016. CiteSpace is a science mapping tool for finding critical points for developing a field or a domain via analyzing trends and patterns in the scientific literature.^20^, ^22^ The node size in each network represents the number of citations obtained by the related references. The ring color around the node represents different years. The thickness of the purple ring reflects the degree of centrality, which is associated with the scientific contribution. Hubs strongly connecting the other two nodes have higher centrality.^23^ Burst keyword detection represents the sudden changes in the events in a certain period. Details for each analysis were described previously.^23^, ^24^

## 3 RESULTS

### 3.1 Bibliometric analysis of publication outputs

The number of publications visually reflects the development trend in a certain field within a period.^22^ A total of 16,264 original and review articles were identified for further bibliometric analysis. The number of publications related to VCI increased yearly, with some fluctuations between 2000 and 2013 (Figure 1A). The difference between 2000 and 2021 (355 versus 1276, respectively) suggests that VCI is increasingly gaining attention worldwide. In total, 191 countries/regions were involved. The United States contributed almost one‐third of all publications (5046, 31.03%), nearly three to four times higher than the following mainland China (1869, 11.49%) and the United Kingdom (1272, 7.82%) (Table 1). The speed of publication increased by fifty times during the last two decades in mainland China (6 in 2000 versus 299 in 2021) (Figure 1A). The top five productive institutions included the Karolinska Institute, University of Toronto, University of California, San Francisco, Mayo Clinic, and the University of Gothenburg. They were distributed in the United States and Europe, contributing 1385 publications (Table 1). These results indicate that the United States and Europe were still in the leading position of VCI research.

**TABLE 1:** Top 10 countries/region and institutions in terms of publications for vascular cognitive impairment

Ranking	Country/Region	Publications	Institution	Publications
1	USA	5046	Karolinska Institute	329
2	Chinese mainland	1869	University of Toronto	293
3	UK	1775	University of California, San Francisco	277
4	Italy	1272	Mayo clinic	246
5	Germany	1100	University of Gothenburg	240
6	Netherlands	1045	University College London	240
7	Canada	978	University of Pittsburgh	234
8	Japan	919	University of California, Davis	233
9	France	865	Johns Hopkins University	230
10	Sweden	845	King's College London	229

### 3.2 Basic knowledge structures of VCI research

#### 3.2.1 Collaborating countries/Regions and institutions in VCI research

A National/regional cooperation analysis was conducted via CiteSpace to reflect the cooperation between countries/regions in the field of VCI. The analysis detected 181 nodes and 821 edges, representing 181 countries/regions contributing to VCI research. The United States, United Kingdom, Japan, France, and Spain were defined as the center of international cooperation and exchange with other countries (purple rings, Figure 1B). Notably, the rings had a short distance from each other, indicating that the collaboration among the nations was relatively close. The assessment of centrality reflects the influence and importance of the nodes in the network, revealing that the United States (0.17) and the United Kingdom (0.17) shared a much greater influence, followed by Japan (0.16) and France (0.16) (Table 2).

**TABLE 2:** Top 10 countries/region and institutions in terms of centrality for vascular cognitive impairment

Ranking	Country/Region	Centrality	Institution	Centrality
1	USA	0.17	National Institute of Aging	0.1
2	UK	0.17	University Of Munich	0.09
3	Japan	0.16	Boston University	0.09
4	France	0.16	University of Florence	0.07
5	Spain	0.12	University of Gothenburg	0.07
6	Germany	0.1	University College London	0.07
7	Canada	0.1	University of California, San Francisco	0.07
8	Scotland	0.1	University of Southern California	0.06
9	Brazil	0.1	University of Melbourne	0.06
10	Australia	0.09	Massachusetts General Hospital	0.06

Also, the analysis of institutional cooperation generated an institution map with 636 nodes and 2215 edges, with a low density (density = 0.011). It indicates that the research groups were relatively scattered in various institutions (Figure 1C). The National Institute of Aging had the leading influence (0.1), followed by the university of Munich (0.09) and Boston University (0.09) (Table 2). Overall, these findings suggest that more scientific collaborations among the institutes are needed.

#### 3.2.2 Analysis of authors and co‐cited authors

**FIGURE 2:**
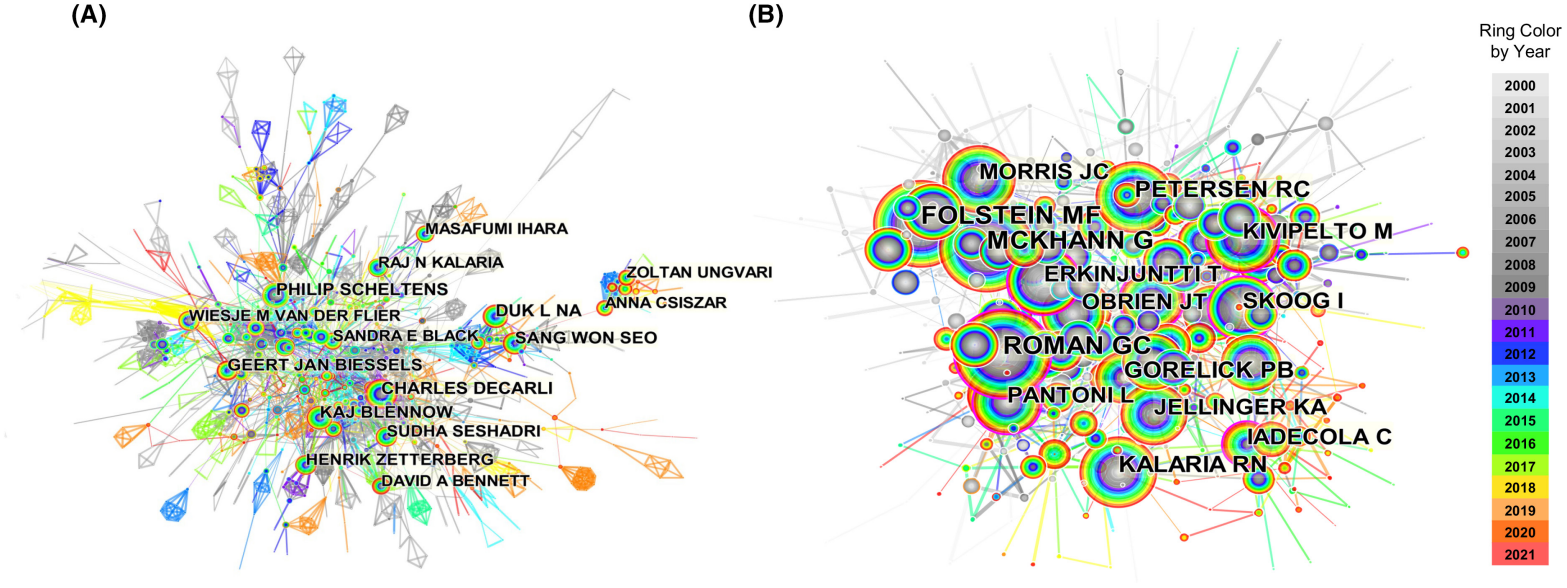
Network map of authors (A) and the co‐cited authors (B) map in vascular cognitive impairment research.

The author collaboration analysis identified 2642 nodes and 6735 edges, which revealed that 2642 authors contributed to 16,264 publications (Figure 2A). The low density (0.0019) suggests that the authors in VCI research are relatively scattered. Among them, Philip Scheltens (155/0.26), Charles Decarli (103/0.13), and Leonardo Pantoni (91/0.22) were the most active authors regarding publication numbers and centrality (Table 3). Philip Scheltens focused on early diagnosis and monitoring of different types of dementia. In particular, his interest was in predicting dementia in patients with mild cognitive impairment via neuroimaging, especially MRI.^25^ Charles Decarli and Leonardo Pantoni also focused on neuroimaging, proposing quantitative measures of SVD in imaging.^8^, ^26^, ^27^, ^28^

**TABLE 3:** Top 10 active authors in terms of publications and centrality for vascular cognitive impairment

Ranking	Publications	Author	Centrality	Author
1	155	Philip Scheltens	0.26	Philip Scheltens
2	103	Charles Decarli	0.22	Leonardo Pantoni
3	97	Leonardo Pantoni	0.13	Charles Decarli
4	91	Duk L Na	0.1	Sandra E Black
5	85	Sang Won Seo	0.05	Giovanni B Frisoni
6	83	Kaj Blennow	0.05	Clifford R
7	83	Geert Jan Biessele	0.04	Frederik Barkhof
8	80	Henrik Zetterberg	0.04	T Erkinjuntti
9	78	Sudha Seshadri	0.04	Ingmar Skoog
10	72	Sandra E Black	0.04	Michael Ewers

We also conducted a co‐cited author analysis to identify the authors of an article and cluster the authors with highly similar research interests. In total, CiteSpace identified 354 nodes and 1267 edges (Figure 2B). Roman GC had the top rank for citation counts (2964) (Table 4), whose research mainly focused on the standardized diagnosis of VCI and its cognition classification consensus.^8^ The other highly co‐cited authors, like Folstein MF (2909), reported that the presence and severity of markers of cerebrovascular pathology are related to the metabolic biomarker homocysteine,^29^ while Mckhann G (2530) found that midlife vascular risk factors are associated with increased risk of dementia.^30^

**TABLE 4:** Top 10 co‐cited authors in vascular cognitive impairment research in terms of co‐citation counts and centrality

Ranking	Co‐citation counts	Cited author	Centrality	Cited author
1	2964	Roman GC	0.18	Erkinjunttit T
2	2909	Folstein MF	0.14	Kivipelto M
3	2530	Mckhann G	0.12	Roman GC
4	1590	Petersen RC	0.12	Pantoni L
5	1464	Kalaria RN	0.11	Skoog I
6	1317	Gorelick PB	0.11	Jack CR
7	1288	Pantoni L	0.10	Iadecola C
8	1255	Erkinjunttit T	0.10	Esiri MM
9	1213	Iadecola C	0.09	Snowdon DA
10	1194	Kivipelto M	0.09	Knopman DS

### 3.3 Overview of research trends and hotspots

#### 3.3.1 Highly co‐cited references and clustering of co‐cited references

Co‐cited publications metric represents the scientific relevance of publications, with each node representing a cited article. With 429 citations, the most co‐cited counts article was the review by Philip B.Gorelick and his colleagues published on *Stroke* in 2011^31^ (Table 5). This scientific statement provided an overview of the evidence on vascular contributions to cognitive impairment and dementia. The authors indicated that there is a need for prospective, quantitative, clinical‐pathological‐neuroimaging studies to improve our knowledge about the pathological alteration and neuroimaging findings in VCI. In addition, future studies are needed to uncover the complex interplay between VCI and AD.^32^ We summarize that the top 10 most co‐cited articles mainly covered the definition, diagnosis, pathobiology and potential treatment of VCI.

**TABLE 5:** Top 10 co‐cited references related to vascular cognitive impairment in terms of co‐citations

Ranking	Co‐citation counts	Cited reference	Representative author (publication year)
1	429	Vascular Contributions to Cognitive Impairment and Dementia: A Statement for Healthcare Professionals From the American Heart Association/American Stroke Association	Philip B. Gorelick (2011)
2	279	Neuroimaging standards for research into small vessel disease and its contribution to aging and neurodegeneration	Wardlaw JM (2013)
3	248	The diagnosis of dementia due to Alzheimer's disease: Recommendations from the National Institute on Aging‐Alzheimer's Association workgroups on diagnostic guidelines for Alzheimer's disease	Guy M. Mckhann (2011)
4	224	The Pathobiology of Vascular Dementia	Costantino ladecola (2013)
5	199	The Lancet International Commission on Dementia Prevention and Care	G Livingston (2017)
6	185	NIA‐AA Research Framework: Toward a biological definition of Alzheimer's disease	Jack CR (2018)
7	180	Vascular dementia	JT O'Brien (2015)
8	180	The global prevalence of dementia: A systematic review and meta‐analysis	Prince M (2013)
9	166	Plasma Homocysteine as a Risk Factor for Dementia and Alzheimer's Disease	S Seshadri (2002)
10	164	Efficacy of galantamine in probable vascular dementia and Alzheimer's disease combined with cerebrovascular disease: a randomized trail	T Eekinjuntti (2002)

**FIGURE 3:**
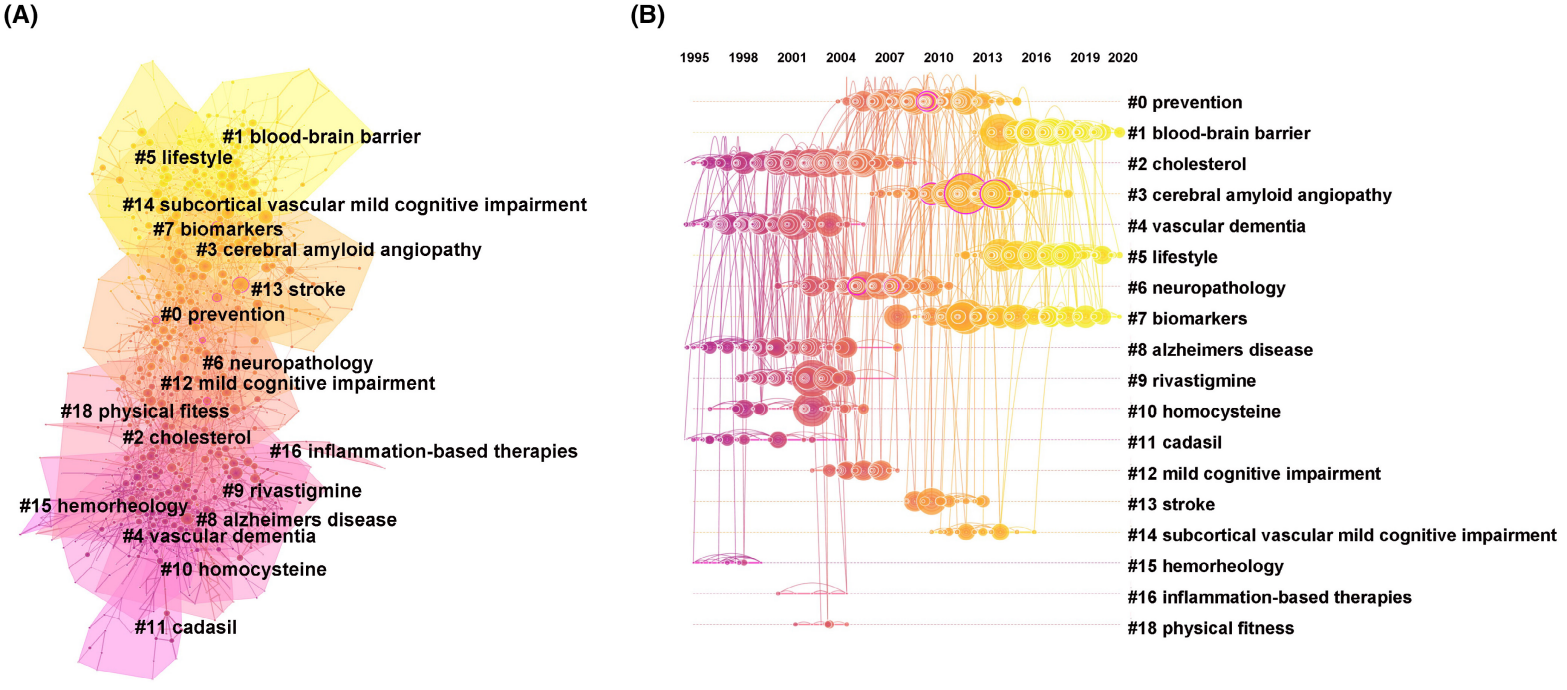
Clustered network map of keywords (A) on vascular cognitive impairment and timeline view of keyword clusters with their cluster‐labels on the right (B).

Clustering of co‐cited references is helpful for excavating the frontiers of the field.^33^, ^34^ Further clustering of these co‐cited references resulted in 1265 nodes, 2466 edges, and 19 main clusters (Figure 3A), with their timelines for each cluster label (Figure 3B). The position of the nodes in these clusters suggests that the pioneering research focus on VCI involves the definition of VCI, prevention, treatments, risk factors, and the potential mechanism of cognitive impairment. The major advantages of these studies were discussed later on.

#### 3.3.2 Cluster analysis of keyword co‐occurrence on VCI hotspots

**FIGURE 4:**
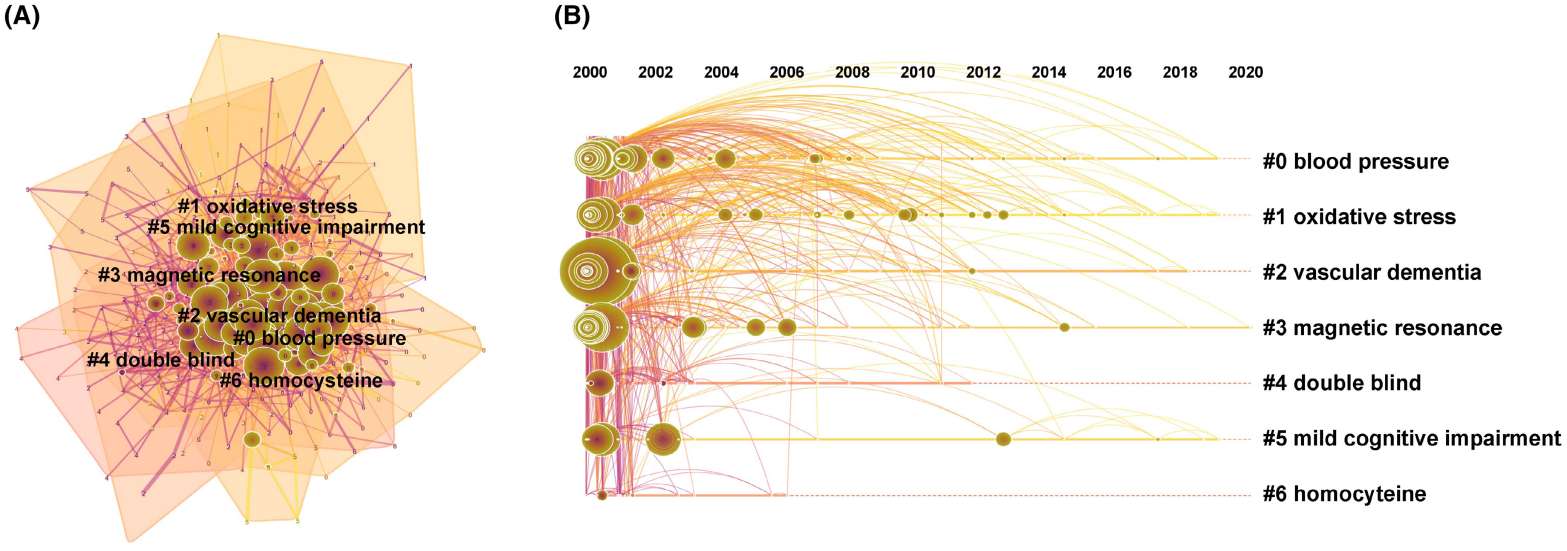
Clustered network map of co‐cited references (A) with their cluster‐labels on the right (B) on vascular cognitive impairment.

Cluster analysis of keyword co‐occurrence on VCI revealed 307 uses of keywords from 2000 to 2021. The analysis showed seven clusters representing the hot topics (Figure 4A) and their timeline (Figure 4B). The position of the nodes represents the time of occurrence. The size of the nodes represents the frequency, which reflects the research hotspots. Unsurprisingly, the most frequent occurrence involved blood pressure (cluster 0). Other clusters were oxidative stress, vascular dementia, magnetic resonance, double‐blind, mild cognitive impairment, and homocysteine. These hot topics included diagnostic methods (magnetic resonance, cluster 3), potential risk factors (blood pressure, cluster 0; oxidative stress, cluster 1), and clinical biomarkers (homocysteine, cluster 6) of VCI. In addition, AD (7568), VaD (4366), and dementia (3122) were the most frequent keywords. AD (0.29), VaD (0.1), and white matter lesion (0.08) were the top‐ranked influencing keywords, predicting keywords co‐occurrence (Table 6).

**TABLE 6:** Top 20 keywords in terms of frequency and centrality in vascular cognitive impairment research

Ranking	Frequency	Keyword	Centrality	Keyword
1	7568	Alzheimer's disease	0.29	Alzheimer's disease
2	4366	Vascular dementia	0.1	Vascular dementia
3	3122	Dementia	0.08	White matter lesion
4	2126	Cognitive impairment	0.08	Oxidative stress
5	2066	Risk factor	0.07	Cerebral blood flow
6	1903	Mild cognitive impairment	0.07	Double blind
7	1381	Brain	0.06	Dementia
8	1365	Risk	0.06	Disease
9	1355	Impairment	0.06	Vascular risk factor
10	1306	Prevalence	0.06	Cerebrospinal fluid
11	1148	Disease	0.06	Blood brain barrier
12	1093	Association	0.06	White matter
13	1086	Diagnosis	0.06	Depression
14	1042	Stroke	0.06	Atrophy
15	1039	Vascular risk factor	0.06	Chronic cerebral hypoperfusion
16	960	Blood pressure	0.05	Risk
17	945	Decline	0.05	Impairment
18	928	Alzheimer's disease	0.05	Stroke
19	924	Population	0.05	Cognitive decline
20	868	Small vessel disease	0.05	Criteria

#### 3.3.3 Keywords and reference citation burst detection

**FIGURE 5:**
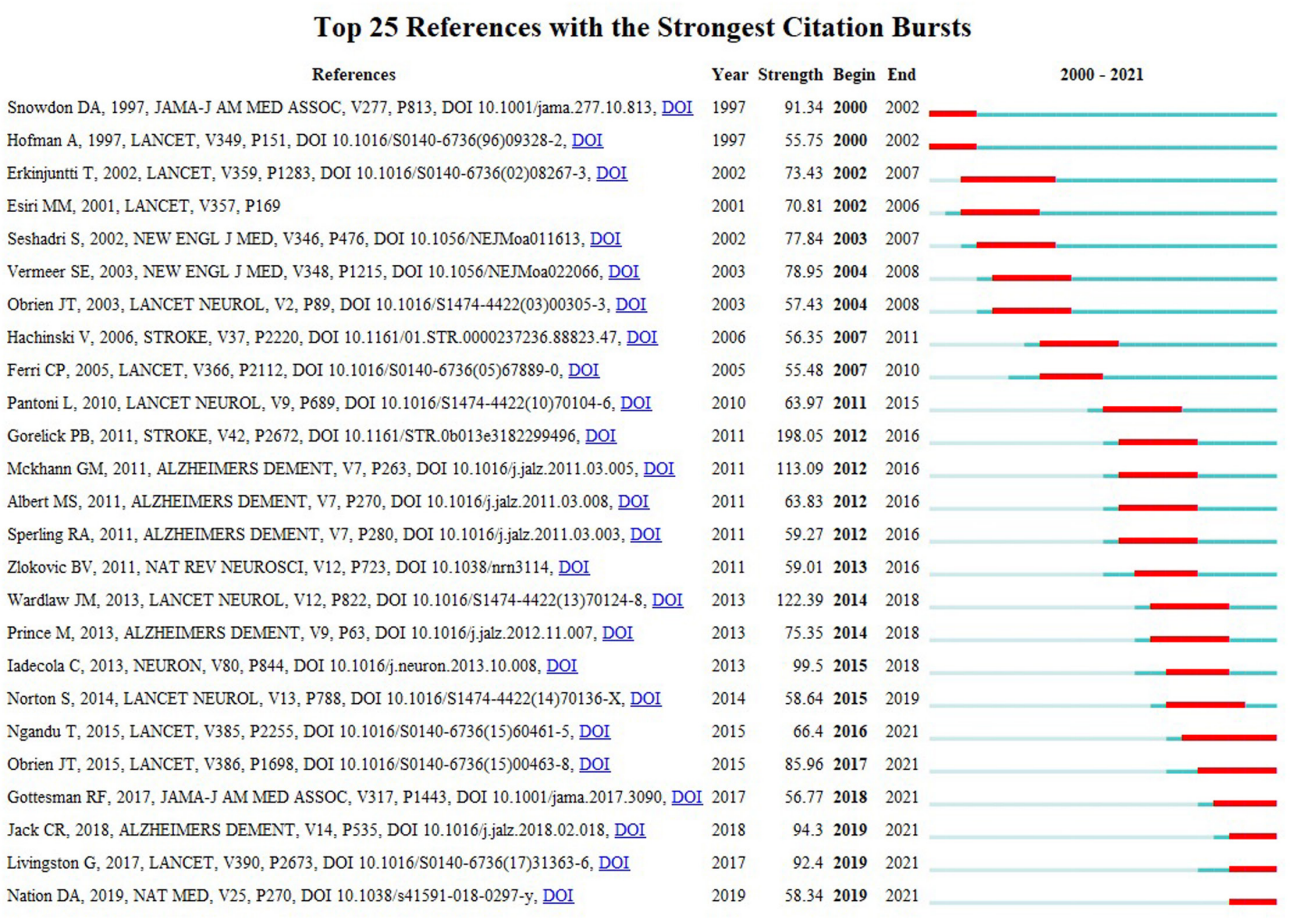
References with the strongest citation bursts in publications on vascular cognitive impairment between 2000 and 2021.

Reference citation burst detection usually revealed a work of great potential or interest and hit a key part of the complex system in the academic field. Paper by Gorelick published at *Stroke* ranked first with the highest burst strength (198.05),^31^ which also had the highest citation numbers mentioned above. Six papers, including Ngandu T, Lancet (66.4), Obrien JT, Lancet (85.96), Gottesman RF, JAMA (56.77), Jack CR, Alzheimers Dementia (94.3), Livingston G, Lancet (92.4) and Nation DA, Nature Medicine (58.34), burst till 2021 (Figure 5). These papers defined that biomarkers, risk factors, together with high quality clinical trial are the hotspots on VCI research.

**FIGURE 6:**
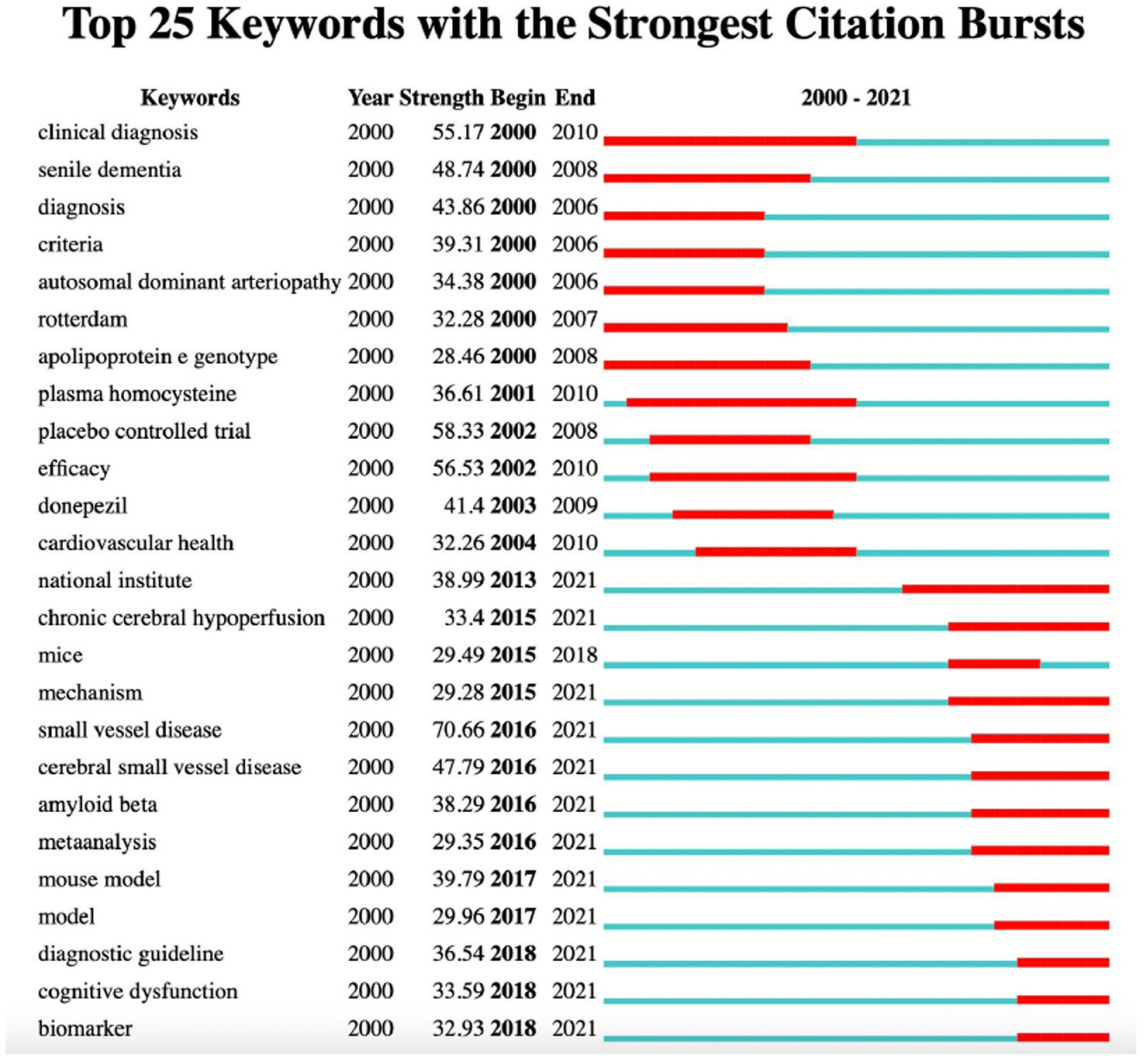
Keywords with the strongest citation bursts in publications on vascular cognitive impairment between 2000 and 2021.

Burst keyword detection was performed to identify the emerging concepts cited frequently over a period of time. Over the past two decades, small vessel disease ranked first with the highest burst strength (70.66), followed by placebo‐controlled trial (58.33), efficacy (56.53), clinical diagnosis (55.17), and senile dementia (48.74) (Figure 6). Only small vessel disease burst from 2016 and continued until 2021, companied with cerebral small vessel disease (47.79), amyloid beta (38.29), meta‐analysis (29.35), and mouse model (39.79), which indicated that these are the current research hotspots.

## 4 DISCUSSION

### 4.1 Primary findings

This study analyzed 16,264 VCI publications from 2000 to 2021 using bibliometric analysis. The overall number of publications discussing VCI is as risen over two decades. The United States was the leading country for both publications and centrality. Besides, publications in mainland China have been growing rapidly. Specifically, from 2013 to 2017, the literature search showed a steady growth. The Karolinska Institute contributed the most publications. Philip Scheltens had the highest influence. The studies mainly focused on prevention, mechanisms, and biomarkers. The clusters of co‐cited publications indicated that studies over the past two decades mainly focused on prevention, treatments, and mechanisms. Keywords analysis suggests that small vessel disease is currently a hotspot in the field of VCI.

### 4.2 Results of the study in context

#### 4.2.1 1. The current academic situation of countries/regions and institutions on VCI research

According to the distributions of countries and institutions, the United States and Europe were undoubtably leading in VCI research. The United States was the leading country regarding both publications and centrality. Japan is the only Asian country in top rank in terms of centrality for VCI. Earlier initiation of VCI research in the United States and Europe led to the present trend. We can also see that publications of mainland China have been rising rapidly in the past two decades. This trend may be closely related to the country's economic growth and the improvement of national health awareness. Further progress will occur in VCI research with the increasing attention of all countries and increased international collaboration. Based on the low density of the cooperation analysis, we propose research institutions should remove academic barriers and communicate to promote the research and development of VCI.

#### 4.2.2 2. Neuroimaging remains gold standard for diagnosis and prevention of VCI

According to the authors' collaboration analysis, the most influential author was Phillp Scheltens, who focused on the early diagnosis of VaD or AD using neuroimaging.^1^, ^35^, ^36^ Charles Decarli and Leonardo Pantoni also focused on neuroimaging. They proposed quantitative measures of SVD in neuroimaging, such as volumetric measurement of WMH or WM fiber structure.^8^, ^26^, ^27^, ^28^ In addition, the keyword analysis found that MRI is also one of the hotspots in the field of VCI. Neuroimaging markers with MRI are the gold standard for the clinical diagnosis of VCI.^7^, ^8^ For example, white matter hyperintensities (WMHs) seen in FLAIR or T2 sequence, microinfarcts seen in diffusion‐weighted imaging (DWI), and microbleeds seen in SWI sequence can help diagnosis and subtype determination of VCI. They are also associated with cognitive impairment and increased risk of stroke and mortality.^37^, ^38^, ^39^ In addition to classical sequences mentioned earlier, novel imaging technology is emerging to measure cerebrovascular injuries. For instance, gadolinium‐enhanced MRI measures contrast agent leakage from the blood plasma to the brain interstitial space and evaluates blood–brain barrier (BBB) integrity. It allows the detection of subtle leakage in aging, SVD, MCI, VaD, and AD.^40^ High‐resolution neuroimaging such as 7 T MRI can identify microinfarcts <1 mm in diameter. New imaging technologies have contributed to the diagnosis and early prevention of VCI. Considering the concept of “what you see is what you get”, more clinical studies and imaging computing methods are needed to expand their application.

#### 4.2.3 3. The major trends and advantages of studies related to VCI

Taken the clustering analysis and burst detection analysis together, we could summarize the emphasis of VCI research was put on definition, prevention, treatment and potential mechanisms. Paper by Gorelick published at *Stroke* ^31^ with the strongest burst citation was undoubtably a watershed on the definition of VCI, which now is defined as “a syndrome with evidence of clinical stroke or subclinical vascular brain injury and cognitive impairment affecting at least one cognitive domain”. The burst citation for this paper ends at 2016 (Figure 5) due to the wide acceptance on the concept of VCI.

“Prevention” was one of the most important clusters after the clustering of co‐cited publications, together with several risk factors. All of the six burst‐till‐now papers^2^, ^41^, ^42^, ^43^, ^44^, ^45^ (Figure 5) talked about prevention strategies and risk factors. Most risk factors for VCI are modifiable; therefore, targeting these risk factors may prevent VCI. VCI can be delayed or even reserved by early prevention. Early in the 1970 s, the multi‐infarcts dementia concept implied that if a stroke is preventable, so should cognitive impairment. Eliminating the seven most common modifiable risk factors of vascular dementia, including obesity, hypertension, diabetes mellitus, high cholesterol, smoking, low level of education, and cardiovascular disease, is estimated to reduce approximately one‐third of vascular dementia cases. Besides, identifying families with hereditary dementia caused by cerebral small vessel disease led to a deeper understanding of VCI.^46^ Notably, studies in patients with cerebral autosomal dominant arteriopathy (CADASIL) have delineated the cognitive profile of pure subcortical ischemic vascular dementia, the neuroimaging correlates of VCI and the therapeutic response to pharmacological interventions.^47^ It was also shown that there is a link between middle‐age cholesterol and obesity, and senile dementia. The BBB limits cerebral deposition of potentially neurotoxic components of plasma. Its permeability may increase in subjects with SVD, VaD, and AD.^6^, ^47^, ^48^ These findings suggest that finding the underlying mechanisms of VCI and prevention strategies is of great clinical significance.

However, it cannot be ignored that there are less large‐scale and high quality clinical trials to access effective methods to prevent VCI or gain ideal therapies on the reduction of cognitive decline. So it is not surprised that trial by Ngandu T^41^ ranked in the top 25 references with the strongest citation bursts (66.4) and burst from 2016 till now. This clinical trial is the first large‐scale, randomized, controlled clinical trial to provide evidence of multidomain assessments, including diet, exercise, cognitive training and other lifestyle interventions, could reduce the risk of cognitive decline. Besides, we conducted the search term of VCI on Clinialtrials.gov and got 171 registered trials, including large‐scale trial (SINGER study conducted in Singapore with estimated 1200 participants), new imaging method trial (7 T MRI on cerebral small vessel disease study conducted in Cambridge) and several new drugs trials. We believe that high quality clinical trial on VCI research would attract more and more attention for the researchers and thus providing effective method or treatment on prevention and treatment of VCI.

Interestingly, small vessel disease (SVD) is the representative burst keyword in recent years. This might as a result of the development of MRI imaging technology and the advance of preclinical studies. Cerebral small vessels, including cerebral end arteries and arterioles, undergo progressive age‐related changes, which alter perfusion and cause microfracts and lacunar infarcts, presenting as cystic lesions <1 cm in diameter.^46^ MRI can detect latent small vessel lesions.^49^ Quantitative measures in imaging have been proposed as the markers of the SVD.^9^, ^49^ Future research on SVD can help explore the neuropathological correlates between small vessel diseases and cognitive impairment and develop new diagnostic and therapeutic interventions.^50^ Meanwhile, the mouse model burst from 2017. Currently, the mouse model of bilateral common carotid artery stenosis (BCAS) is widely used to reproduce cognitive decline induced by chronic cerebral hypoperfusion in patients. It is a well‐established animal model for studying VCI.^51^ The preclinical studies provided new mechanistic insight and discovered biomarkers and therapeutic strategies through genetic manipulation, pharmacological inhibition, and other methods, which could not be applied in clinical studies.^52^ Drug discovery had little progress in VCI and VaD.^48^ Extensive preclinical studies are needed to identify “druggable” targets based on pathologic pathways involved in VCI and VaD. Taken together, both preclinical and clinical efforts on the topic of neuroimaging, risk factors detection for prevention, and pathobiology are helpful for VCI research.

## 5 STRENGTH AND LIMITATIONS

To our knowledge, this bibliometric analysis is the first study identifying the trends and hotspots in the scientific research related to VCI. By including 16,264 original and review articles within two decades, we generated a more comprehensive overview of VCI research, which determines future studies' direction.

This study also has some weaknesses. First, the study design was based on publications in the WoSCC database; therefore, many relevant publications in other databases such as Medline, SCOPUS, and the Cochrane library were not included. As CiteSpace was developed based on the WoSCC database, we selected it for further study. Second, the CiteSpace analysis is based on the number of citations and structural variation and information fitness theory,^53^ which can be affected by many factors and cannot fully reflect the real quality of articles. For example, review articles are usually with higher citations. We did not separate articles and reviews in the analysis for that we expected to got an overview of the entire field of VCI without a node loss. Similarly, we did not separate preclinical studies and clinical studies in this analysis for the same reason. Further detailed bibliometric analysis could be applied on the subtype of VCI research. Third, we noticed that AD accounted for a large proportion of keywords analysis because previous studies did not sufficiently differentiate between AD and VaD. In addition, the prevalence of mixed cerebrovascular and AD pathology is high. The visualized analysis based on literature undoubtedly helps scholars understand the research subjects, research hotspots in VCI.

## 6 CONCLUSION

This bibliometric analysis visualized the overall structure of the research on VCI. The current research trends and hotspots can be a guide for future studies. The study found that the small vessel disease and its mechanisms were the current hotspots in VCI research. Efforts on neuroimaging, risk factors detection, or pathobiology will be helpful for the prevention and improvement of VCI.

## CONFLICT OF INTEREST

The authors declare there is no conflict of interest.

## Data Availability

The date generated from this study are available upon request from the corresponding author.
